# Mortality after osteoporotic hip fracture: incidence, trends, and associated factors

**DOI:** 10.1186/s13018-019-1226-6

**Published:** 2019-07-04

**Authors:** Olalla Guzon-Illescas, Elia Perez Fernandez, Natalia Crespí Villarias, Francisco Javier Quirós Donate, Marina Peña, Carlos Alonso-Blas, Alberto García-Vadillo, Ramon Mazzucchelli

**Affiliations:** 1 Department of Rehabilitation, Hospital Universitario Fundación Alcorcon, Alcorcon, Madrid Spain; 2 Department of Rheumatology, Hospital Universitario Fundación Alcorcon, Alcorcon, Madrid Spain; 3 Department of Clinical Investigation, Hospital Universitario Fundación Alcorcón, Alcorcon, Madrid Spain; 4 Health Center La Rivota (Alcorcon), Alcorcon, Madrid Spain; 5 Emergency Department, Hospital Universitario Clínica Puerta de Hierro de Majadahonda, Majadahonda, Madrid Spain; 6 Department of Rheumatology, Hospital Universitario La Princesa, Madrid, Spain

**Keywords:** Mortality, Incidence, Epidemiology, Osteoporosis, Femur fracture, Hip fracture

## Abstract

**Background:**

It is known that mortality after hip fracture increases compared to the general population; the trend in mortality is a controversial issue.

The objective of this study is to examine incidence, trends, and factors associated with mortality in patients with osteoporotic hip fractures.

**Methods:**

This is a retrospective cohort study that uses the Registry for Hospital Discharges of the National Health System of our hospital. Patients older than 45 having an osteoporotic hip fracture between 1999 and 2015 were identified. Demographic data and comorbidities were obtained. A survival analysis was performed (Cox regression and Kaplan-Meier). Incidence rate, standardized death rate (SDR), trend (Poisson regression), and risk (hazard ratio) were calculated.

**Results:**

During 1999–2015, in our hospital, there were a total of 3992 patients admitted due to osteoporotic hip fracture. Out of these 3992 patients, 3109 patients (77.9%) were women with an average age of 84.47 years (SD 8.45) and 803 (22.1%) were men with an average age of 81.64 years (SD 10.08). The cumulative incidence of mortality was 69.38%. The cumulative mortality rate for 12 months was 33%. The annual mortality was 144.9/1000 patients/year. The 1-year mortality rate increased significantly by 2% per year (IRR 1.020, CI95% 1.008–1.033). The median overall survival was 886 days (CI95% 836–951). The probability of mortality density for a period of 10 years following a hip fracture was 16% for women and 25% for men (first 90 days). The SDR was 8.3 (CI95% 7.98–8.59). Variables that showed statistically significant association with mortality were aged over 75, masculine, institutionalization, mild to severe liver disease, chronic kidney disease, COPD, dementia, heart failure, diabetes, the Charlson Index > 2 , presence of vision disorders and hearing impairment, incontinence, and Downton scale.

**Conclusions:**

For the last 17 years, an increase of mortality for patients with hip fracture and a higher mortality rate in men than in women were observed. Institutionalization combined with comorbidities is associated with a higher mortality.

**Electronic supplementary material:**

The online version of this article (10.1186/s13018-019-1226-6) contains supplementary material, which is available to authorized users.

## Mini abstract

The purpose of this study is to analyze mortality trend after having a first episode of osteoporotic hip fracture and factors associated with mortality over a long period of time (17 years). It was observed that the 1-year mortality rate increased 2% per year during the study period. Institutionalization combined with comorbidities is associated with a higher mortality.

## Introduction

Osteoporotic hip fracture is a major health problem due to increased mortality, morbidity, and functional impact in these patients (only 30–40% of these patients recover their previous functional status) [1, 2], as well as the economic cost for the National Health System. In addition, it is expected to increase as life expectancy increases.

Increased mortality after hip fracture has been widely reported. The cumulative mortality after 1 year of a hip fracture occurrence ranges between 20 and 40% [3–7] with higher mortality rates in men than in women [8–10]. After having a hip fracture, the mortality risk from any other cause increases between five and eight times, and although it decreases over the first years, excess mortality stays higher than mortality for the general population for a period of at least 10 years [10, 11].

Literature describes different factors associated with mortality, among them were age, masculine gender, dementia, heart pathology, institutionalization, comorbidity, and change of residence or rehabilitation programs carried out after hip fracture [12–14].

One aspect of mortality associated with hip fracture, and seldom researched, is the trend of mortality rates over a long period of time.

The purpose of this study is to analyze mortality, as well as factors associated and trends in mortality over time in patients with osteoporotic hip fracture in an urban municipality of Spain.

## Material and methods

Retrospective observational cohort study for the area covered by the Hospital Universitario Fundación Alcorcon from January 1, 1999, until December 31, 2015.

### Introduction

Alcorcon is in the metropolitan area of Madrid in Spain. It is a clear example of a dormitory town since half of its population works in Madrid. Its population pyramid is typical of an aging population. The Hospital of Alcorcon is the only public hospital servicing this district and all the hip fractures occurring in the area. Until 2011, the sphere of influence of the Hospital included 18 municipalities (17 were rural and 1 urban), among them was Alcorcon city, with a total population of 273,703 inhabitants. However, from 2012, the hospital only attended one municipality: Alcorcon city with 167,136 inhabitants.

### Data source and definitions

Records on hip fractures (HFx) were obtained from the admissions’ database of this hospital. All hospitals in Spain use this database which collects the minimum basic data set (MBDS) of each patient admitted to any Spanish hospital: reasons for admission, procedures, and diagnosis codified in accordance with International Classification of Diseases 9 (ICD-9). We ascertained all hip fracture cases occurred in persons 45 years old or older from ICD-9 codes 820.0 to 820.9 in the study years. All cases that occurred to subjects aged 45 years or older from ICD-9 820.0 to 820.9 codes during that period of study were identified, including both first and second instances of fracture. Thanks to a recent work published by our team and where the same database was used, we know that 7.5% of all fractures included in that period correspond to second fractures [15]. Only osteoporotic fractures were taken into consideration. As an example, pathologic fractures (ICD-9 733.1–733.19), metastasis, metabolic diseases such as Paget’s disease (M89.9), femur fractures of different location (821.0–821.9), or acetabular pelvis fractures (808.0–808.9) as well as fractures due to traffic accident (V87.9) were excluded. Data derived from readmissions were removed. An admission in the same center within the first 30 days after discharge due to the same medical reason was considered a readmission. Patients having a fracture within that period had a follow-up until the end of the study (December 31, 2015). The MBDS was queried to check whether the patient had died or had been readmitted for a different reason after the fracture. Losses to follow-up or deceased patients were cross-checked with death certificates obtained from the National Death Index or INDEF, by its Spanish acronym (https://indef.msssi.es/indefWeb/loginAction.do). Second fractures were excluded from the survival analysis, and only the date of the first fracture was considered for analysis. In this study, with the objective of facilitating the comparison with previous studies, only population over 45 years of age were included.

The following data were collected at the moment of the first fracture: sex, age, place of residence (nursing home or own home), type of fracture (intra- or extracapsular), time elapsed until surgery, and time of hospitalization. Additionally, the following data on comorbidity (identified by ICD code) were recorded: dementia, diabetes mellitus, obesity, neoplasm, mild to severe liver disease (defined as evidence of portal hypertension: ascites, esophageal varices, or encephalopathy), chronic obstructive pulmonary disease, skin sores, cardiovascular disease (ischemic cardiopathology, brain stroke, congestive heart failure, and peripheral artery disease). Charlson Index calculation was performed based on comorbidity data. Additional data were collected from the Nursing Assessment Form on admission. This questionnaire started to be used in our center in 2010, so only data from the last 5 years of study were collected. During this period of 5 years, 1154 patients were included, and out of this total, the form was completed for 810 patients. Data collected by this questionnaire consisted of vision disorder and hearing impairment, urinary and/or fecal incontinence, all the Norton scale items plus its final score that quantifies the risk of bed sores, the Downton scale, technical aid to the patient’s evolution, and habitual treatment.

To calculate standardized mortality ratios (SMRs), the population and mortality in Madrid were used as a reference. Data were obtained from the Statistical Institute of the Community of Madrid (http://www.madrid.org/iestadis).

### Statistical analysis

The follow-up period covers the time between the dates of hospital admission, due to hip fracture, and patient’s death. Three 4-year initial periods and one 5-year final period were defined for the analysis.

The annual mortality rate was calculated after the occurrence of an osteoporotic hip fracture per 100,000 inhabitants in people older than 45 and by sex, the numerator being the cases of death after first hip fracture registered in the MBDS and the denominator being the population at risk (those who had a first hip fracture and were still alive).

The cumulative rate of mortality was calculated for 1, 3, 6, 12, and 36 months over the time of study (17 years), global, and by sex. The probability of mortality density was also analyzed during a period of 10 years following a hip fracture. Specific mortality rates by age and sex, based on the time between fracture and death or end of study, were compared to rates of expected mortality by age and sex in the Madrid population (standardized mortality ratios or SMRs). Assuming a Poisson distribution, statistical significance and the respective 95% confidence intervals (CI95%) were calculated.

Kaplan-Meier survival analysis was used to compare survival time between the different periods and by age and sex. Bivariate and multivariate Cox regression analyses were performed to analyze differences between patients and factors associated with mortality. Results were expressed as hazard ratios (HR) with their corresponding CI95% values.

Change in the annual rates of death after hip fracture was analyzed by generalized linear models (GLM) with Poisson distribution or negative binomial distribution in case of over-dispersed data. Models were used to estimate the incidence rate ratio of the variables at 1, 3, and 12 months with CI95%; the *p* value of association was noted as a result of the trend test. The significance level was established at 1% to account for the use of aggregated data. To allow for a clearance period, year 1999 was excluded from the analysis.

Patients’ clinical and demographic characteristics were described. Quantitative data were described by their median and standard deviation or by median and interquartile range (IQR). Qualitative data were described by counts and percentage. The relative frequency of the different variables for each period was also analyzed in order to find out if they have changed over the study period.

The statistical analysis was carried out with IBM SPSS 24.0 and Stata 14 software.

## Results

During the study period (1999–2015), there were a total of 3992 patients admitted due to osteoporotic hip fracture. Out of these 3992 patients, 3109 patients (77.9%) were women with an average age of 84.47 years (SD 8.45) and 803 (22.1%) were men with an average age of 81.64 years (SD 10.08). Moreover, 86.2% were patients of age over 75 years.

The majority of patients (73.9%) were living in their own homes and not institutionalized before the fracture occurrence. Among institutionalized patients, 81.95% were women. The average Charlson Index was 1.08 (SD 1.50), and in women, it was 0.70 (SD 1.14). Out of the total amount, 7.4% of patients had a Charlson Index above 2 (men 12.1% out of the total of men and women 6.1% out of the total of women).

### Cumulative mortality

Global cumulative mortality over the 17 years of study was 69.38% (634 men and 2136 women). Cumulative mortality rate for 1, 3, 6, 12, and 36 months after fracture was 9.2%, 17.4%, 24.6%, 33%, and 56%, respectively, being higher in men than in women (men 13.7%, 25%, 32.7%, 43.3%, and 65.6%; women 7.9%, 15.7%, 22.3%, 30%, 53.2%).

### Mortality rates

Table 1 shows crude mortality rate adjusted by age, sex, and year of study. Annual mortality rate in population older than 45 years was 144.9/1000 patients/year (173.70 in men and 138.11 in women). Table 2 shows crude mortality rate by sex and age-adjusted.

**Table 1:** Crude and age-adjusted mortality rate by year for both sexes

Year	Hip fractures	Population at risk	Deaths	Crude rate/1000 patient-year	Age-adjusted rate/1000 patient-year	CI95%	
1999	190	190	39	202.07	208.69	144.96	355.57
2000	227	378	76	200.53	212.30	165.16	270.71
2001	237	539	75	135.44	150.20	116.95	190.17
2002	244	708	101	138.42	149.16	120.20	183.22
2003	265	872	142	162.84	174.71	146.27	207.41
2004	280	1010	146	142.57	151.77	127.38	179.61
2005	295	1159	194	167.39	174.70	150.39	201.93
2006	268	1233	177	142.74	148.01	126.69	172.04
2007	258	1314	204	155.25	158.82	137.63	182.77
2008	292	1402	237	169.04	171.75	150.40	195.82
2009	282	1447	210	145.13	147.46	128.15	169.01
2010	266	1503	223	148.37	149.55	130.54	170.65
2011	293	1573	223	141.13	142.51	124.37	162.68
2012	181	1531	218	142.39	142.56	124.25	162.95
2013	119	1432	169	117.32	118.79	101.49	138.31
2014	151	1414	147	103.96	105.00	88.70	123.43
2015	144	1411	189	133.95	133.95	115.53	154.47
1999–2015	3992	19,116	2770	144.90

**Table 2:** Crude and age-adjusted mortality rate by sex and year

	Hip fractures	Population at risk	Deaths	Crude rate/1000 patient-year	Age-adjusted rate/1000 patient-year	CI95%	
Women							
1999	153	153	28	183.01	186.82	118.51	326.13
2000	177	302	63	208.61	224.13	169.38	293.99
2001	183	422	53	125.59	139.94	102.73	188.26
2002	192	561	74	131.91	141.15	109.32	179.84
2003	220	707	112	158.42	176.29	144.05	214.96
2004	222	817	109	133.41	150.59	123.30	182.75
2005	228	936	153	163.46	174.76	147.38	206.60
2006	206	989	127	128.41	135.91	113.05	162.60
2007	205	1067	153	143.39	150.12	126.99	177.06
2008	236	1150	183	159.13	169.62	145.95	196.90
2009	215	1182	154	130.29	135.11	114.44	159.03
2010	212	1240	180	145.16	147.71	126.68	171.52
2011	225	1285	173	134.63	138.09	118.22	160.79
2012	128	1240	171	137.90	143.76	123.26	167.07
2013	90	1159	138	119.07	117.93	98.71	139.93
2014	114	1135	117	103.08	100.49	82.73	121.03
2015	103	1121	148	132.02	125.78	105.88	148.34
1999–2015	3109	15,466	2136	138.11			
Men							
1999	37	37	11	297.30	295.79	147.56	591.88
2000	50	76	13	171.05	169.08	87.10	307.21
2001	54	117	22	188.03	196.58	125.59	309.22
2002	52	147	27	183.67	192.08	127.12	291.36
2003	45	165	30	181.82	175.55	116.41	256.60
2004	58	193	37	191.71	173.93	118.76	249.59
2005	67	223	41	183.86	180.97	129.09	251.78
2006	62	244	50	204.92	195.37	143.97	264.84
2007	53	247	51	206.48	209.05	155.70	277.16
2008	56	252	54	214.29	182.87	134.51	244.29
2009	67	265	56	211.32	201.32	151.36	265.03
2010	54	263	43	163.50	163.04	118.56	222.57
2011	68	288	50	173.61	183.14	135.40	244.04
2012	53	291	47	161.51	139.93	100.27	191.67
2013	29	273	31	113.55	127.14	88.17	180.23
2014	37	279	30	107.53	123.00	85.54	171.93
2015	41	290	41	141.38	164.95	121.62	218.70
1999–2015	883	3650	634	173.70			

### Trend in mortality

**Fig. 1:**
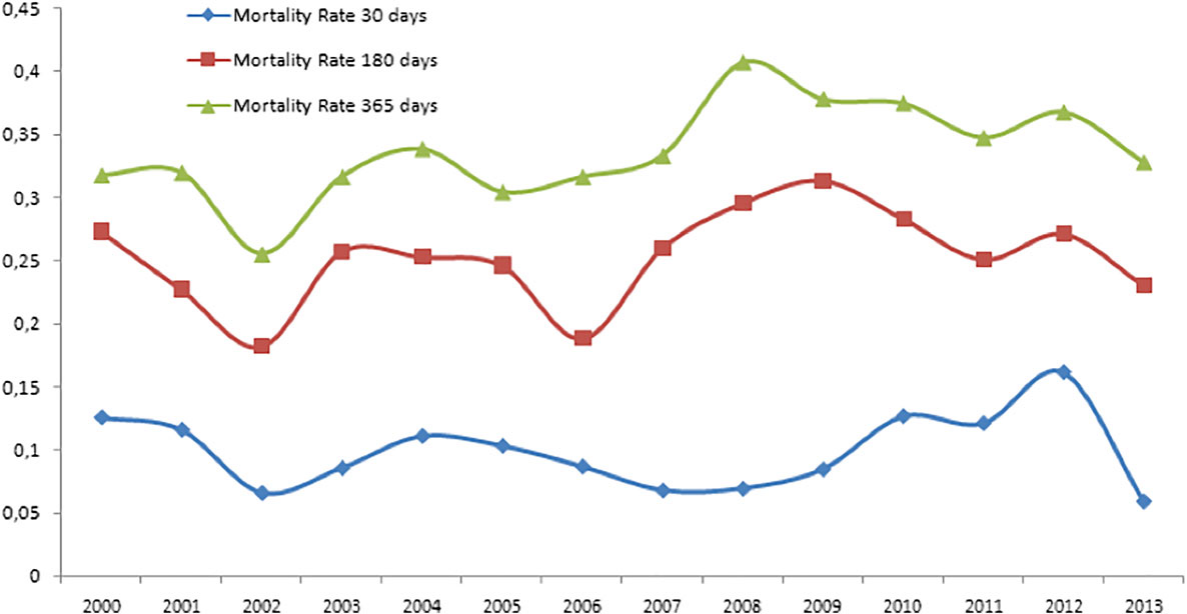
Trend in mortality rate for 30, 180, and 365 days

Through the use of GLM, it is observed that 1-year mortality rate increased 2% per year in a significant way (IRR 1.020, CI95% 1.008–1.033). One-month mortality rate remained stable without increasing or decreasing (IRR 1.020, CI95% 0.967–1.050) and the same happened for 6-month mortality rate (IRR 1.016, CI95% 0.997–1.035) (Fig. 1).

### Probability of mortality density

**Fig. 2:**
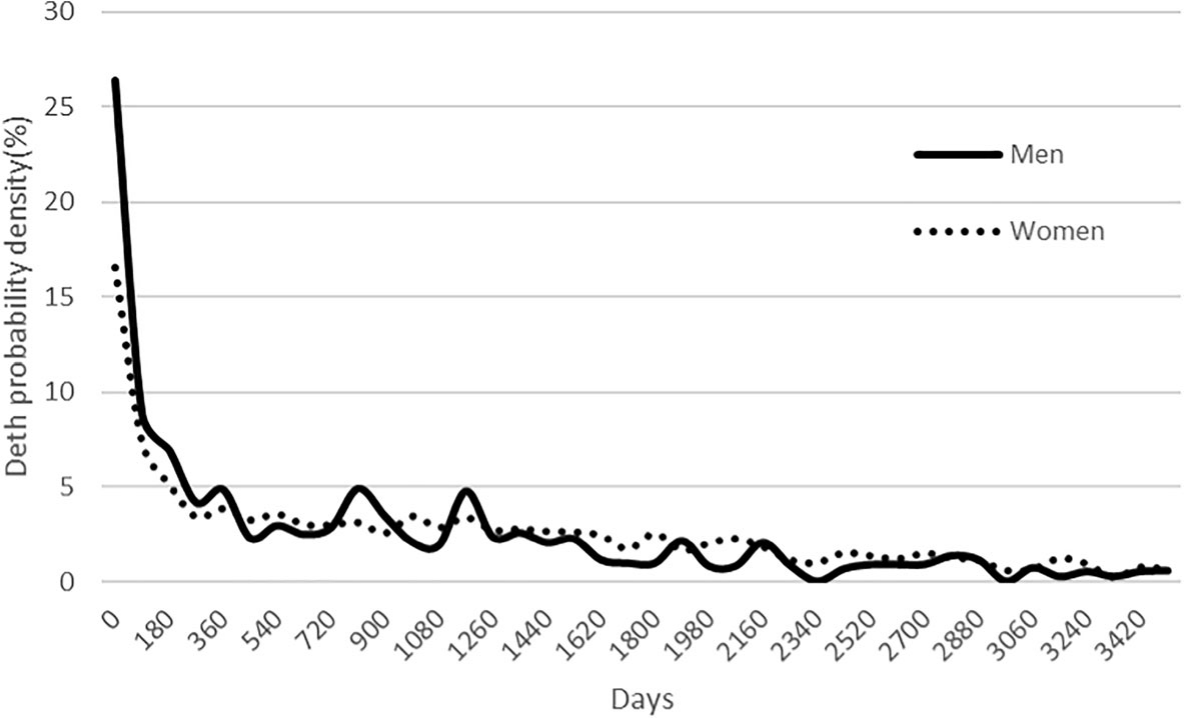
Probability of mortality density after hip fracture in the next 10 years, by sex

Figure 2 shows probability of mortality density for a period of 10 years following the hip fracture occurrence. It is around 16% of women and 25% of men in the first 90 days, falling rapidly in the first year and stabilizing around 2% every 90 days in the following years.

### Standardized mortality

Regarding general mortality in Madrid, the standardized mortality ratio (SMR) in the 17 years of study was 8.3 (CI95% 7.98–8.59) with very similar values in men (8.03; CI95% 7.43–8.47) and women (8.77; CI95% 8.41–9.14).

### Factors associated to mortality after hip fracture occurrence

The bivariate regression Cox analysis (Table 3) shows association between mortality and the following items: age (IRR 1.05, CI95% 1.04–1.06), masculine gender (IRR 1.32, CI95% 1.21–1.44), institutionalization (IRR 1.48, CI95% 1.36–1.60), Charlson Index 2 (IRR 1.98, CI95% 1.73–2.26), mild or severe hepatic disease (IRR 3.25, CI95% 2.17–4.86), kidney failure (IRR 1.87, CI95% 1.60–2.18), heart failure (IRR 2.50, CI95% 2.07–3.00), chronic obstructive pulmonary disease (IRR 1.46, CI 95% 1.30–1.64), diabetes (IRR 1.11, CI95% 1.02–1.2), or dementia prior to the fracture (IRR 1.46, CI95% 1.30–1.62). From the factors collected in the Nursing Assessment Form, those associated were hearing impairment (IRR 1.552, CI95% 1.18–1.97) and previous vision disorders (IRR 1.50, CI95% 1.11–2.03), urinary incontinence (IRR 1.53, CI95% 1.25–1.88) and fecal incontinence (IRR 1.66, CI95% 1.32–2.07), and Downton scale total score (IRR 1.65, CI95% 1.32–2.08).

**Table 3:** Risk factors associated with mortality

	Hazard ratio	CI95% for Exp(B)	
		Lower	Upper
Men	1.323*	1.213	1.443
Charlson > 2	1.984*	1.738	2.264
> 75 years old	2.482*	2.173	2.835
Age	1.055*	1.049	1.060
Institutionalization	1.476*	1.362	1.600
Diabetes	1.120*	1.024	1.225
Obesity	0.909	0.726	1.136
Sever to moderate liver disease	3.249*	2.172	4.862
COPD	1.465*	1.303	1.648
Cerebrovascular disease	1.136	.973	1.326
Bedsores	1.377	.877	2.162
Peripheral artery disease	1.785*	1.237	2.575
Myocardial infarction	1.711*	1.399	2.094
Intracapsular fracture	0.953	0.884	1.026
Heart failure	2.498*	2.077	3.004
Neoplasm	1.881*	1.511	2.341
Dementia	1.456*	1.309	1.621
Renal insufficiency	1.873*	1.603	2.187
Hearing impairment**	1.529*	1.186	1.970
Vision disorders**	1.503*	1.110	2.037
Urinary incontinence**	1.537*	1.251	1.889
Fecal incontinence**	1.660*	1.329	2.075
Antiaggregant therapy**	1.173	.918	1.498
Anticoagulant therapy**	1.320	.969	1.798
Downton scale**	1.657*	1.321	2.080
Norton scale 5–11**	0.469*	0.356	0.619

*p < 0.05

**Data exclusively obtained from subgroup of patients with a Nursing Assessment Form on admission

**Fig. 3:**
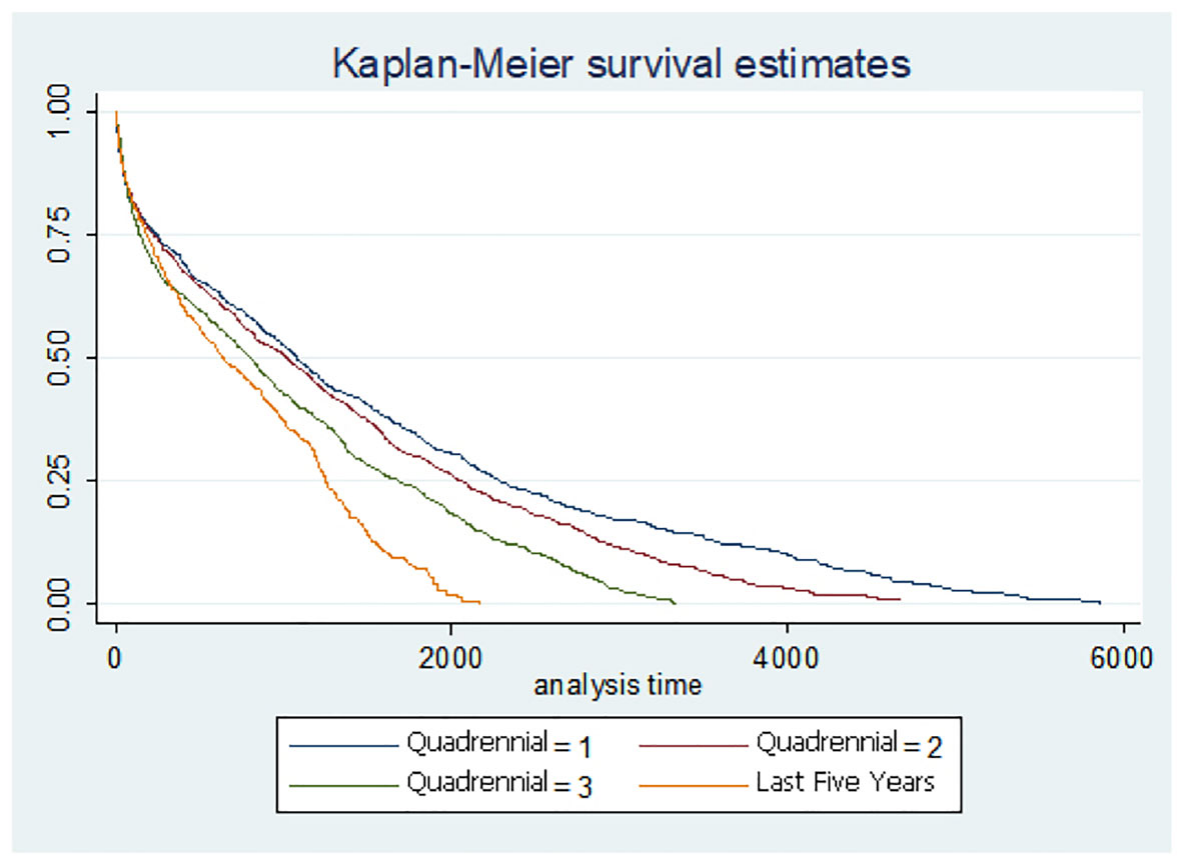
Survival analysis by periods of study

In the survival analysis, men die more frequently and earlier than women after hip fracture (Additional file 1: Figure S1). In the analysis by periods, the trend is that mortality is also earlier (Fig. 3).

## Discussion

Numerous studies proved that the mortality risk after hip fracture is increasing [10, 11, 16, 17]. This increase in mortality is not exclusive to hip fracture, and it is also associated with vertebral fractures [18, 19] and practically all major osteoporotic fractures, such as humerus fracture [20, 21], pelvis fracture [22, 26], distal end of femur fracture[22], and rib fracture [22, 26], and at all ages [18, 19]. Moreover, a second fracture results in an additional increase in mortality [18].

In this study, mortality in patients with hip fracture has been analyzed over a long period of time (1999–2015). This allowed for research into trends in mortality rates, which is currently a controversial topic. The main finding of this study is that the trend in 1-year mortality rate has increased 2% per year during the period of study. Other findings corroborate well-known results obtained from other studies: in the first place, mortality in patients with hip fracture increases a lot compared to the general population; secondly, mortality rate is higher in men than in women; thirdly, death risk is very high right after the fracture occurrence (it drops over the first year and remains stable afterwards); fourthly, institutionalization combined with comorbidity is associated with a higher mortality.

A few studies presented results on trends in mortality after osteoporotic hip fracture. Some of them, from different nationalities, show that mortality remained stable over the years [16, 23, 24]. Other studies described small increases in excess mortality due to hip fracture in specific population groups (elderly women) [10], and others described a variable reduction, although moderate in general, in mortality rates over time [25–28]. Being a study that covers a long period of time, this study allows us to analyze trends in mortality in patients with hip fracture. The study observes a growing trend of mortality of 2% per year at 1 year, while short-term mortality remains stable (1 to 6 months). We believe that the observed increase in mortality rate is due to a growing proportion of men over women occurred over the years of the study period, and men, as is explained below, have a higher mortality rate. While in the first period of the study (1999–2002) men had 21.5% of all fractures, in the final period (2011–2015), this figure increased up to 25.7%. Another factor, probably having an impact on this raise, is the increase in comorbidity observed over the period of study. While in the first period (1999–2002) 5.3% of patients had a Charlson Index over 2, in the final period (2011–2015), it increased up to 11.1%.

In accordance with different studies, cumulative 1-year mortality after hip fracture varies between 20 and 40% [5, 29, 30] with higher rates of mortality observed in men than women [8, 31]. In previous studies done in Spain, 1-year mortality has been placed around 21–23% [12, 13] and varies widely depending on the regions [32]. In this study, a cumulative 1-year mortality of 30% in women and 43% in men has been obtained, which is clearly higher than data obtained from previous studies. The difference between results obtained in this study and previous studies can possibly be found in the methodology used. In this study, we have searched for patients’ deaths through the national database of death certificates, while in the rest of studies this was not done.

In this study, the standardized mortality index for the whole period of study (17 years) has been analyzed in comparison with the global mortality in Madrid based on data obtained from the National Institute of Statistics (INE, by its Spanish acronym). We obtained an 8 SMR. This finding is similar to those of other authors working with series such as the Forsen study [33], the Johnell study [34], or the Fransen study [35]. Forsen et al. [33] found a 1-year mortality risk of 9 in subjects aged between 50 and 74 years, 5.1 for subjects aged between 75 and 85 years, and 5.7 for subjects older than 85. Johnel et al. [34] found a 1-year mortality risk of 10.2 in men and 9.1 in women. Fransen et al. [35, 40] found a 2-year mortality risk of 7.18 in women.

Epidemiological studies have consistently proved that men present a higher risk of mortality [8, 9]. This study, as other studies did, found a higher risk of mortality in men than in women. Masculine sex presents a 30% increase in death risk compared to women. In contrast to other studies that do not find that excess mortality in men compared to women is due to comorbidities [36], this study observed that men present higher comorbidity, measured by the Charlson Index which would justify this increase of mortality.

Likewise, the study observes, as other studies do [7], the fact that death risk is very high immediately after hip fracture and that this risk rapidly drops over the first year, stabilizing with a slight trend downwards over the following years (Fig. 2).

Lastly, the role of comorbidities as a factor increasing risk mortality is controversial [37]. In our study, the risk factors most strongly associated with mortality (HR > 2) were moderate-severe liver disease, to be older than 75 years, and heart failure. Other risk factors also associated with mortality (HR between 1 and 2) were male, Charlson index greater than 2, diabetes mellitus, living in a nursing home, COPD, myocardial infarction, dementia, and renal failure. It has been hypothesized that comorbidities play a major role as a cause of mortality after fracture. In fact, several studies [12, 38], as this study did, found an association between the severity of comorbidities and mortality risk. In a study based on Medicare USA data, after adjustment by comorbidities, the risk of mortality dropped significantly over the early post-fracture period (first 6 months) and disappeared completely over the later post fracture period of follow-up (over 6 months) [37]. Due to this dramatic drop of excess mortality with adjustment of state of health, the authors concluded that the majority of early deaths and all late deaths were not attributable to hip fracture. In a different Spanish study, the severity of comorbidities was also found to be associated with an increase of mortality after vertebral fracture [37]. Nevertheless, not all studies found an association between comorbidity and post-fracture mortality. In the Study Osteoporotic Fractures (SOF), the adjustment by comorbidity did not affect the relationship between mortality risk and any of the examined fractures [39]. Similar findings were obtained in other studies [40]. This study is groundbreaking as we had the chance to analyze the data collected from a Nursing Assessment Form on admission for a subgroup of 810 patients. This form collects specific data on the patient’s quality of life before admission, such as vision and hearing capacity, presence of urinary incontinence, fecal incontinence, Downton scale (measuring risk of falling), and Norton scale (measuring risk of developing bedsores). In this study, it was found that, with the exception of Norton scale total score, all these factors are associated with risk of death. It seems that we can conclude that a previous bad quality of life is associated with a higher mortality after hip fracture. Unfortunately, the absence of data on established treatments does not allow us to assess with certainty whether different treatments have been taken or avoided in these groups with higher risk. It is striking that among the associated factors are institutionalized and older people, that is, the more fragile patients who sometimes have to stabilize before proceeding with surgical treatment, delaying one of the known factors that increase mortality. In relation to the increase in mortality in males, the associated comorbidities that sometimes require prior stabilization is also one of the main causes of surgical delays. In daily practice, knowing the most vulnerable subgroups forces us to maximize therapeutic care in these subgroups with a higher risk of mortality.

This study also presents some limitations, among them were those typical of a retrospective study that uses administrative databases as main source, and these limitations may cause a loss of data. Additionally, since it is a large-scale population and a long period of study, some variables could not be obtained from all patients (for example, date of surgery or data collected on the Nursing Assessment Form on admission which was only implemented in our center for the last few years of study). Time delay between the fracture and surgery could not be observed. Important variables for this profile of patient, i.e., the use of different treatments such as bisphosphonates and other therapeutic methods, could not be examined since our database lacks this type of data. Another limitation of our work, it is that which refers to the use of HR in retrospective cohort studies. The use of the HR for causal inference is not straightforward even in the absence of unmeasured confounding, measurement error, and model misspecification. Endowing a HR with a causal interpretation is risky for 2 key reasons: the HR may change over time, and the HR has a built-in selection bias. Nevertheless, this study presents some strength. It is a series with a large number of patients (3992) that includes all osteoporotic hip fractures treated in a tertiary hospital. For this reason, we think that this study is very representative of the urban population of the center of Spain, and it was carried out over a long period of time (17 years). Additionally, numerous variables were collected, and some of them, related to mortality after hip fracture, have not been studied until now, such as Downton or Norton scales.

In conclusion, in this study, we found that 1-year mortality has increased over the last 17 years, and we corroborate specific aspects of mortality that were established in other previous epidemiologic studies, i.e., that mortality in patients with hip fracture increased highly compared to the general population, that mortality risk is higher in men than in women, that death risk is very high immediately after fracture and drops over the first year remaining stable afterwards, and that institutionalization combined with comorbidity are associated with higher mortality. Additionally, some factors related to quality of life before hip fracture are identified, i.e., vision disorders, hearing impairment, incontinence, or Downton scale, which are associated with an increase in mortality for these patients.

## Additional file

Additional file 1: **Figure S1.** Supplementary material. Survival analysis by sex. (DOCX 153 kb)

## Data Availability

The datasets generated during and/or analyzed during the current study are not publicly available due to the protected data from the Hospital records but are available from the corresponding author on reasonable request. Some data like population census are available at the National Statistical Institute of Spain (www.ine.es). Some data such as the Spanish minimal hospital common data or CMBD are available at the URL https://pestadistico.inteligenciadegestion.mscbs.es.

## Abbreviations

CI95%: 95% confidence interval
COPD: Chronic obstructive pulmonary disease
HFx: Hip fracture
HR: Hazard ratio
ICD-9: International Classification of Diseases 9
INDEF: Índice Nacional de Defunción
IRR: Incidence rate ratio
MBDS: Minimum basic data set
SD: Standard deviation
SDR: Standardized **d**eath **r**ate
