# Supplementary material for: Mortality after osteoporotic hip fracture: incidence, trends, and associated factors
Source: J Orthop Surg Res. 2019 Jul 4;14:203. doi: 10.1186/s13018-019-1226-6 (PMC6610901; doi:10.1186/s13018-019-1226-6)
Supplement: Supplementary file 1 — Figure S1. Supplementary material. Survival analysis by sex. (DOCX 153 kb) [file 13018_2019_1226_MOESM1_ESM.docx]

**SUPPLEMENTARY MATERIAL**

Figure 1, supplementary material. Survival analysis by sex.


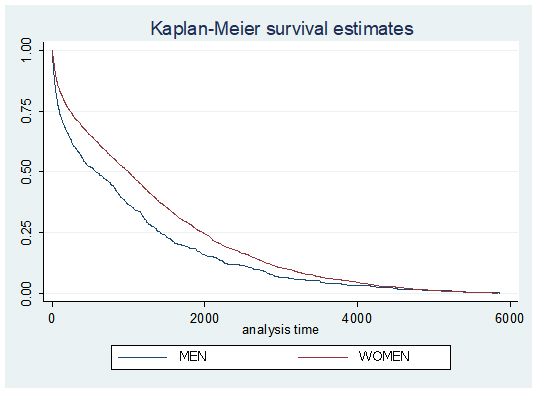


**DECLARATIONS**

- **Ethics approval and consent to participate**

The Clinical Ethics Committee of the Hospital Universitario Fundacion Alcorcón approved the study

- **Consent for publication**

Not applicable

- **Availability of data and material**

The datasets generated during and/or analysed during the current study are not publicly available due to be protected data from the Hospital records but are available from the corresponding author on reasonable request. Some data like poblational census are available at the National Stadistical Institute of spain ([www.ine.es](http://www.ine.es)). Some data as the spanish (Minimal hospital common data or CMBD are available at the URL http://pestadistico.inteligenciadegestion.msssi.es

- **Competing interests**

The authors declare that they have no competing interests

- **Funding**

Ther was no funding in the development of the article

- **Authors' contributions**

OGI and RM wrote the paper. OGI, RM and NCV designed  the research. EPF and CAB did the main data analysis. CAB also helped in the figures. FJQD and MP collaborated in the bibliographical research process and contributed with the CMBD (hospital data) process

- **Acknowledgements**

The authors thank the staff of the Research Unit of the Spanish Society of Rheumatology for their support in the editing and translation of the manuscript. To my good friend Caligula, who faithfully accompanies me in my research work.
